# Rapid Visual Detection of Cyprinid Herpesvirus 2 Based on One-Tube RPA-CRISPR/Cas12a System

**DOI:** 10.3390/vetsci13090970

**Published:** 2026-09-16

**Authors:** Jiaqi Li, Wen Zou, Yuping Tang, Mingyang Liu, Yongjie Liu, Yuhao Dong

**Affiliations:** MOE Joint International Research Laboratory of Animal Health and Food Safety, College of Veterinary Medicine, Nanjing Agricultural University, Nanjing 210095, China; li_jiaqi@stu.njau.edu.cn (J.L.);

**Keywords:** cyprinid herpesvirus 2, RPA, CRISPR-Cas12a, fluorescence, visual detection

## Abstract

Cyprinid herpesvirus 2 (CyHV-2) is a highly lethal pathogen responsible for substantial economic losses in farmed cyprinids worldwide. However, rapid and field-deployable diagnostic tools remain limited. Here, we developed a one-tube recombinase polymerase amplification (RPA)-CRISPR/Cas12a-based fluorescence assay for the visual detection of CyHV-2. The system integrates RPA pre-amplification targeting the highly conserved helicase gene *hphS*, Cas12a-mediated specific recognition and collateral cleavage of a fluorophore-quencher-labeled ssDNA reporter, and visual readout under a portable blue light transilluminator. After optimizing the concentrations of primers, crRNA, Cas12a and the reaction time, the detection limit of this method reached 1 copy/μL, and it showed no cross-reaction with other common aquatic pathogens. Clinical evaluation using 100 tissue samples demonstrated 100% concordance with quantitative real-time PCR (qPCR). This method does not require expensive equipment and can be completed within approximately one hour. This RPA-CRISPR/Cas12a platform offers a rapid and specific solution for on-site CyHV-2 surveillance, especially suitable for aquaculture environments with limited resources, and is expected to have broader applications in the detection of aquatic pathogens.

## Simple Summary

Cyprinid herpesvirus 2 (CyHV-2) is a highly lethal virus that causes significant economic losses to farmed guppies and goldfish worldwide. Rapid and simple diagnostic methods are needed for on-site monitoring in the aquaculture industry. In this study, we developed a one-tube visual detection method for CyHV-2 by combining recombinase polymerase amplification (RPA) with the CRISPR/Cas12a system. The assay targets the highly conserved *hphS* gene of CyHV-2 and produces a visible green fluorescence signal under a portable blue light transilluminator, eliminating the need for complex instruments. The method achieved a detection limit of 1 copy/μL of DNA sample and showed no cross-reactivity with other common aquatic viruses. Testing on 100 clinical samples from diseased fish yielded results that were 100% consistent with quantitative real-time PCR (qPCR). The entire process, from DNA extraction to result readout, can be completed within approximately 1 h. This simple, sensitive, and equipment-flexible method provides a practical tool for rapid CyHV-2 surveillance, particularly in resource-limited aquaculture farms.

## 1. Introduction

Cyprinid herpesvirus 2 (CyHV-2), also known as the goldfish herpesvirus or the herpesic hematopoietic necrosis virus (HVHN), is the main cause of fatal diseases in farmed carp species worldwide [1]. The disease spreads rapidly among susceptible fish species, especially in densely-crowded farming environments such as ponds and recirculating water systems. So far, CyHV-2 has been reported in multiple countries across Asia, Europe, and North America, causing large-scale deaths of cultured goldfish (*Carassius auratus*) and crucian carp (*Carassius carassius*), with mortality rates of up to 90–100% [2,3]. Therefore, early surveillance of CyHV-2 is a critical measure for controlling the outbreak of the disease.

Currently, the traditional gold standard for CyHV-2 diagnosis is virus isolation, followed by PCR or serological tests for confirmation [4]. However, this method is time-consuming and not suitable for large-scale screening or rapid on-site diagnosis. Immunodiagnostic techniques like enzyme-linked immunosorbent assay (ELISA) and immunocytochemistry have been developed for detecting CyHV-2 [5,6]. However, these antibody-based methods can cross-react with other herpesviruses, affecting their reproducibility and reliability.

In recent years, molecular biology techniques have revolutionized pathogen detection, especially with the emergence of polymerase chain reaction (PCR) technology. Quantitative real-time PCR (qPCR) and droplet digital PCR (ddPCR) have enhanced detection accuracy for aquatic pathogens [7,8]. However, these methods still rely on expensive thermal cycling equipment, specialized reagents, and trained personnel, which limits their application in the field or in resource-limited environments. Isothermal amplification technologies, such as recombinase polymerase amplification (RPA), are another option, which does not need thermal cycling instruments [9,10].

The CRISPR-Cas (Clustered Regularly Interspaced Short Palindromic Repeats/Related Proteins) system has recently been applied to portable nucleic acid testing, including SHERLOCK [11] and DETECTR [12], which have demonstrated remarkable diagnostic utility. In particular, after RNA-guided binding to the target DNA, Cas12a exhibits a non-selective cleavage activity for single-stranded DNA (ssDNA), resulting in the degradation of non-target ssDNA molecules. This substance has been used for signal amplification and visualization through fluorescent reporter genes, where the single-stranded DNA probe labeled with fluorescein-quencher breaks down and generates detectable fluorescence signals [13,14]. The integration of isothermal amplification with CRISPR-Cas12a has facilitated the development of highly sensitive, specific, and field-deployable diagnostic systems for diverse pathogens, including viral, bacterial, and parasitic agents [15,16]. For aquatic disease surveillance, several CRISPR-based platforms have been reported for fish viral pathogens. Lu et al. [17] developed a CRISPR-Cas12a-based assay for the detection of mandarin fish ranavirus (MRV), achieving a detection limit of 1 copies/μL. Similarly, Megarani et al. [18] established a CRISPR-Cas12b-based method for tilapia lake virus (TiLV) detection, demonstrating robust performance in clinical samples. However, the combination of RPA with CRISPR-Cas12a and a simple fluorescence-based visual readout for CyHV-2 detection has not yet been fully optimized. Such an integrated platform could leverage the rapid amplification of RPA, the high specificity and sensitivity of CRISPR-Cas12a, and the visual simplicity of fluorescence detection, providing a comprehensive solution for on-site CyHV-2 diagnosis.

**Figure 1:**
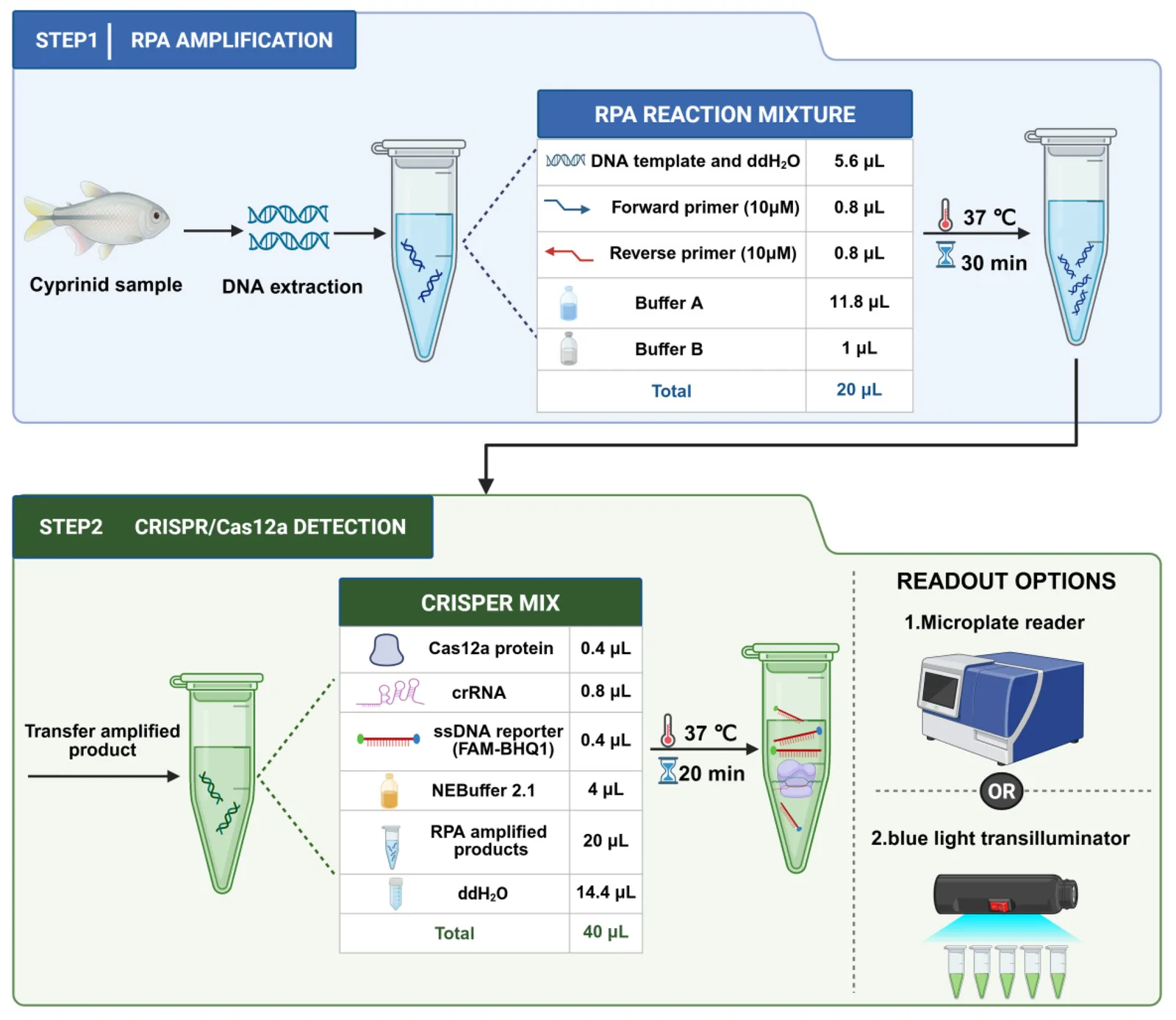
Schematic of the RPA-CRISPR/Cas12a platform for CyHV-2 detection.

In this study, we developed an RPA-CRISPR-Cas12a-based detection method for CyHV-2 (Figure 1). This innovative platform integrates RPA pre-amplification targeting the conserved *hphS* gene of CyHV-2 to enhance sensitivity, CRISPR-Cas12a-mediated specific recognition, and visual fluorescence readout using a portable blue light transilluminator. The detection limit of this platform reaches 1 copy/µL, showing no cross-reaction with other common aquatic pathogens, and was successfully applied to clinical samples. This method provides a rapid, specific, and equipment-flexible diagnostic tool for CyHV-2 surveillance.

Genomic DNA extracted from fish clinical samples was subjected to RPA followed by CRISPR/Cas12a reaction, with fluorescence detection via microplate reader or portable blue light transilluminator. RPA: recombinase polymerase amplification. FAM: fluorescein amidite. BHQ1: Black Hole Quencher 1.

## 2. Materials and Methods

### 2.1. Sample Collection and DNA Extraction

The CyHV-2 (YZ-01 strain, GenBank: MK260012.1) used in this study was isolated from diseased goldfish. Viral nucleic acids of cyprinid herpesvirus 3 (CyHV-3), spring viremia of carp virus (SVCV), and grass carp reovirus (GCRV) were obtained from laboratory-maintained viral stocks. Clinical samples, including gill, kidney, and spleen tissues, were collected from a total of 100 goldfish and crucian carp from three aquaculture farms in Jiangsu Province, China. The samples included 50 diseased fish samples (confirmed positive for CyHV-2 by qPCR), 46 healthy fish samples (qPCR-negative) and 4 samples from asymptomatic carriers (qPCR-positive without clinical signs). Tissue samples were homogenized in 1 mL of phosphate-buffered saline (PBS) using a TissueLyser (Qiagen, Hilden, Germany). Following centrifugation at 10,000× *g* for 5 min, 200 μL of the supernatant was used for subsequent nucleic acid extraction.

### 2.2. Nucleic Acid Extraction

Total viral nucleic acid was extracted from clinical samples and virus stocks using the Viral DNA/RNA Extraction Kit (Omega Bio-tek, Norcross, GA, USA) according to the manufacturer’s instructions. The extracted nucleic acids were eluted in 50 μL of nuclease-free water and stored at −80 °C until use.

### 2.3. Construction of the Positive Plasmid

The *hphS* gene (locus_tag = CyHV2_058) from CyHV−2 was PCR-amplified using viral genomic DNA as the template. Primers are listed in Table 1. The 50-µL amplification mixture contained 25 µL of 2 × Rapid Taq Master Mix, 3 µL of template DNA, 1 µL of each forward and reverse primer (10 µmol/L), and 20 µL of nuclease-free water. The amplified products were ligated at the A-terminal using the DNA tailing kit (Takara, Dalian, China), then inserted into the pMD19-T vector (Takara), and subsequent transformation into competent *E. coli*. The recombinant clones were identified by Sanger sequencing. The verified construct was named pMD-19T-hphS and was stored at −20 °C.

### 2.4. RPA Reaction

RPA assays were performed using the DNA Isothermal Rapid Amplification Kit (Amp-Future, Changzhou, China). Three primer pairs are provided in Table 1. The RPA reaction mixture contained 11.8 μL of buffer A, 1 μL of buffer B, 0.8 μL of each forward and reverse primers (10 μM), 2 μL of DNA template, and 3.6 μL of nuclease-free water to a final volume of 20 μL. For each RPA run, a negative control (NC) and a no-template control (NTC) were included to monitor amplification efficiency and detect potential contamination. To prevent cross-contamination, all RPA operation steps were performed in a dedicated pre-amplification area using filter-tipped pipettes. Additionally, an anti-airborne aerosol barrier is employed throughout the entire process. The reaction mixture was incubated at 37 °C for 30 min in a water bath. The amplified products were confirmed by 2% agarose gel electrophoresis.

### 2.5. crRNA and ssDNA Preparation

Three crRNAs were designed to target *hphS* gene of CyHV-2. The sequences of crRNAs are provided in Table 1. Candidate crRNA sequences were selected based on the presence of a 5′-TTTN-3′ protospacer adjacent motif (PAM) upstream of the target sequence. The crRNA sequences and the ssDNA fluorescence reporter (5′-FAM-TTTATTT-BHQ1-3′) were synthesized by Tsingke Biotech Co., Ltd. (Beijing, China).

### 2.6. Optimization of the One-Tube RPA-CRISPR/Cas12a Detection System

First, the cleavage efficiency of the three crRNAs was evaluated. The RPA-CRISPR/Cas12a reaction mixture contained 0.4 µL of Cas12a (New England Biolabs, Ipswich, MA, USA), 0.8 µL of crRNA, 4 µL of 10× NEBuffer 2.1, 0.4 µL of ssDNA probe, 20 µL of RPA-amplified products, and 14.4 µL of sterile distilled water to a total volume of 40 μL. In every CRISPR/Cas12a reaction, a negative control and a no-template control were included to validate the specificity and reliability of the cleavage reaction. The fluorescence signal was monitored in real-time at 37 °C for 30 min using a Spark^®^ multifunctional microplate reader (Tecan, Shanghai, China) with excitation at 485 nm and emission at 535 nm. All experiments were conducted with a fixed 80-fold manual gain to ensure consistency of the results. The raw fluorescence units (FU) were used directly without additional normalization, as the negative control consistently yielded baseline FU values (~200–500 RFU), and the positive control provided a consistent maximum signal. Endpoint fluorescence values were taken at 20 min for statistical analysis. For classification of samples as positive or negative, a run-specific threshold was defined as the mean fluorescence of negative controls (NC) + 3 × Standard deviation (SD) of negative controls. When the endpoint FU value of the sample was equal to or higher than this threshold, it was considered positive; samples below this threshold were considered negative. To account for the variability between different experiments, this threshold would be recalculated for each individual experiment. The crRNA with the highest cleavage efficiency was selected for further experiments. To establish a one-tube RPA-CRISPR/Cas12a detection platform, the CRISPR/Cas12a mixture (0.4 µL Cas12a protein, 0.8 µL crRNA, 4 µL of 10 × NEBuffer 2.1, 0.4 µL of the reporter and 14.4 µL of nuclease-free water) was loaded into the cap of the tube. The assembled tube was then incubated at 37 °C for 20 min. Afterwards, a brief centrifugation was applied, followed by vortexing to merge the cap-contained CRISPR/Cas12a mix with the RPA product. Checkerboard titration was performed to optimize the concentrations of Cas12a and crRNA. Five concentration levels (1.2, 1.0, 0.8, 0.6, and 0.4 µM) for each component were tested, with fluorescence intensity as the readout. The ssDNA reporter concentration was also optimized across a range of concentrations (1, 3 and 5 µM). Finally, the optimal reaction time for both RPA and CRISPR/Cas12a cleavage was determined. The optimized reaction conditions were then used for all subsequent sensitivity, specificity, and clinical sample testing.

### 2.7. Sensitivity and Specificity Analysis

To determine the limit of detection (LOD) of the RPA-CRISPR/Cas12a system, a recombinant plasmid containing the CyHV-2 *hphS* gene (pMD-19T-*hphS*) was constructed. The plasmid concentration was measured using a NanoDrop spectrophotometer, and the copy number was calculated. The plasmid was serially diluted 10-fold to final concentrations ranging from 1 × 10^5^ to 1 × 10^0^ copies/μL. The LOD was evaluated using both fluorescence microplate reader and visual detection under a portable blue light transilluminator. All visual fluorescence images were acquired under strictly standardized conditions using a digital camera Canon EOS 80D (Canon, Tokyo, Japan) mounted on a fixed stand at a constant distance of 20 cm from the samples, with fixed camera settings (ISO 800, aperture f/5.6, shutter speed 1/30 s, auto white balance). The portable blue light transilluminator (470 nm) was used as the sole light source, and all images were captured in a darkened room. For each sensitivity run, a negative control (nuclease-free water) and a no-template control were included to validate the assay performance. The specificity of the assay was determined using extracted nucleic acids from CyHV-2, CyHV-3, SVCV and GCRV, each at a concentration of approximately 200 ng/μL. Nuclease-free water served as the negative control. All specificity experiments included a positive control (pMD-19T-*hphS*), a negative control and a no-template control to confirm that no non-specific cleavage occurred. A strong fluorescence signal, detected by both the microplate reader and visual inspection, was considered a positive result.

### 2.8. Clinical Sample Testing

To evaluate the clinical performance of the developed assay, a total of 100 tissue samples from diseased goldfish and crucian carp were tested. Genomic nucleic acids were extracted from each sample using the Viral DNA/RNA Extraction Kit as described above. All samples were tested in parallel using the newly developed RPA-CRISPR/Cas12a fluorescence method and a validated quantitative real-time PCR (qPCR) assay targeting the CyHV-2 *hphS* gene, which served as the reference standard. In each clinical test, a positive control, a negative control, and a no-template control were included to ensure the reliability of the results.

### 2.9. Statistical Analysis

All statistical analyses were performed using GraphPad Prism (version 9.0.2, GraphPad Software, La Jolla, CA, USA). Data are presented as mean ± standard deviation (SD) from at least three independent experiments (n = 9 per group). One-way analysis of variance (ANOVA) was used to compare fluorescence intensity means among multiple independent groups, followed by Tukey’s honestly significant difference (HSD) post-hoc test for pairwise comparisons. The assumptions of normality and homogeneity of variances were verified using the Brown-Forsythe and Bartlett’s tests, respectively. *p* value < 0.05 was considered statistically significant. For visual detection under the portable blue light transilluminator, no statistical analysis was performed as this provides a qualitative binary readout (positive/negative).

## 3. Results

### 3.1. Screening and Optimization of RPA Primers for CyHV-2 Detection

**Figure 2:**
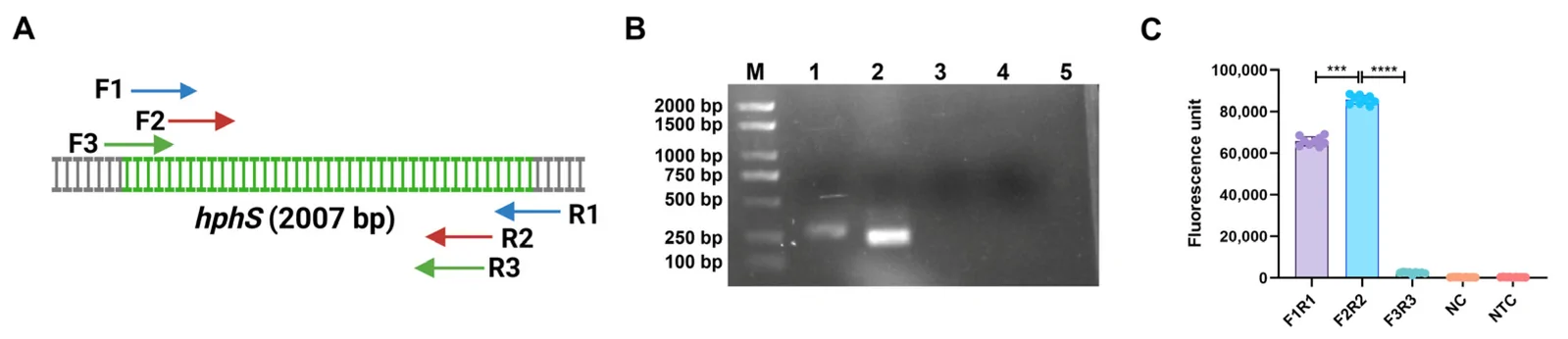
Screening of RPA primers for CyHV-2 detection. (**A**) Schematic diagram of the CyHV-2 *hphS* gene target region showing the approximate locations of the three designed primer pairs. (**B**) Agarose gel electrophoresis analysis of RPA products using the different primer pairs. M: DL2000 DNA marker; Lane 1: F1R1; Lane 2: F2R2; Lane 3: F3R3. Lane 4: NC; Lane 5: NTC. (**C**) Fluorescence signals from CRISPR reactions triggered by each RPA primer set. Data represent mean ± SD from three biological replicates, each with three technical replicates. *** *p* < 0.001, **** *p* < 0.0001. NC: negative control (nuclease-free water); NTC: no-template control.

The efficiency of RPA is critically dependent on primer design, which directly governs amplification kinetics, specificity, and detection sensitivity. The *hphS* gene of CyHV-2 is highly conserved among different isolates and serves as an ideal target for CyHV-2 detection. Based on the sequence of *hphS* gene, three primer pairs were designed following RPA primer design principles (Figure 2A), and their exact target positions within the *hphS* gene are shown in Figure S1. The amplification products were analyzed by agarose gel electrophoresis, revealing a single band at the expected molecular weight for all primer pairs. The F2/R2 primer pair demonstrated the highest amplification efficiency and strongest band intensity with no non-specific products (Figure 2B). Consistent with this result, the F2/R2 primer set exhibited the highest fluorescence signal among all combinations (Figure 2C). Collectively, these results confirm that F2/R2 is the optimal primer pair for reliable and efficient amplification in the following assays.

### 3.2. Optimization of CRISPR-Cas12a Reaction Parameters

**Figure 3:**
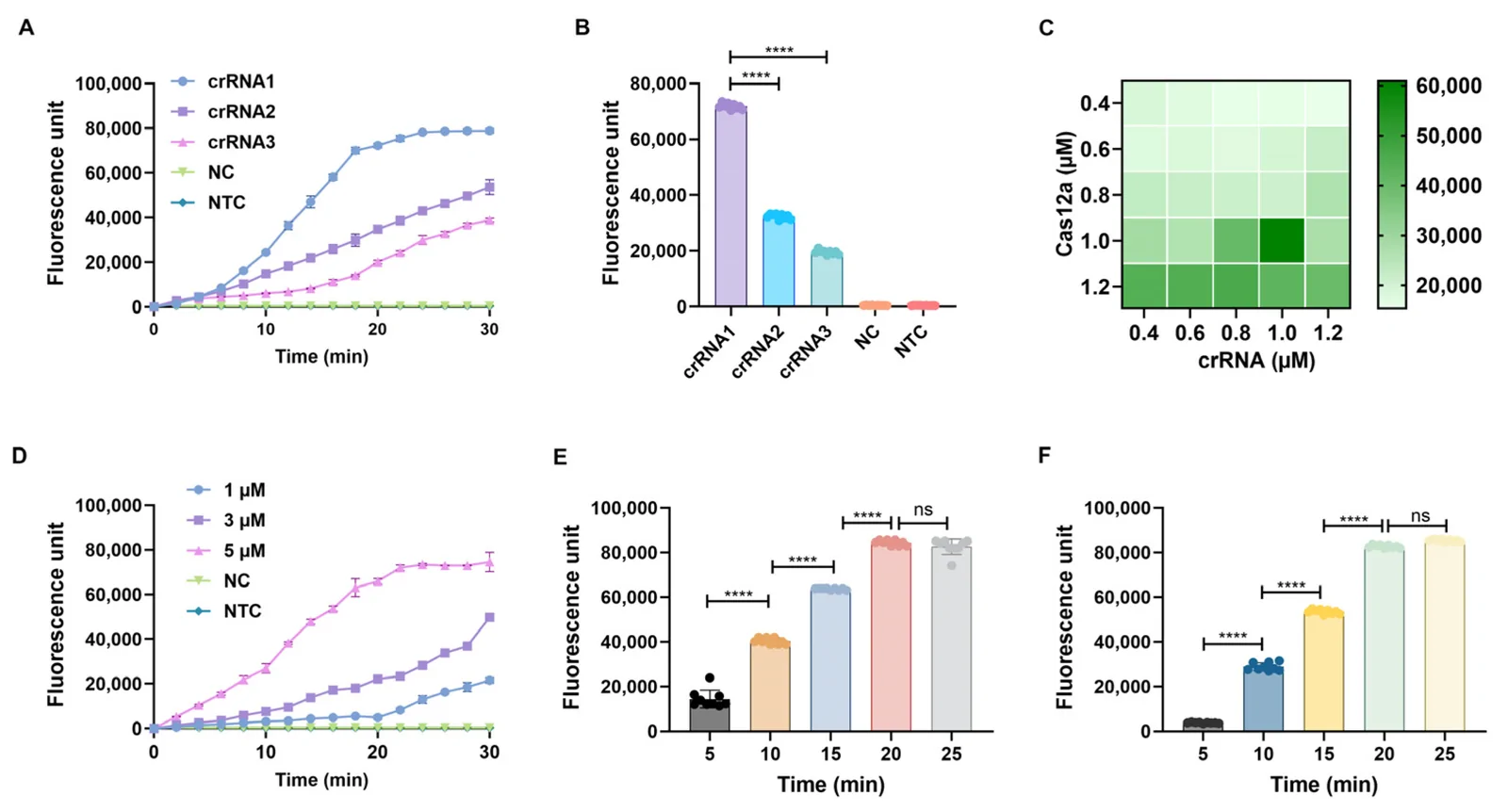
Optimization of RPA-CRISPR/Cas12a detection system. (**A**) Fluorescence curves of three crRNAs designed for the *hphS* gene. (**B**) Fluorescence values of three crRNAs measured at 20 min. (**C**) Checkerboard titration optimization of Cas12a protein and crRNA concentrations. (**D**) Fluorescence determination of optimal ssDNA reporter concentration across a dilution series. (**E**) Optimal RPA incubation time determined within a 1-h CRISPR reaction. (**F**) Optimal CRISPR incubation time determined within a 1-h RPA reaction. Data represent mean ± SD from three biological replicates, each with three technical replicates. **** *p* < 0.0001. ns, not significant. NC: negative control (nuclease-free water); NTC: no-template control.

To maximize the sensitivity of the fluorescence-based detection, the key parameters of the CRISPR-Cas12a reaction system were optimized using a microplate reader for real-time fluorescence monitoring. Firstly, the *hphS* gene from the CyHV-2 genome was amplified by PCR and cloned into the pMD-19T vector to generate the pMD-19T-*hphS* plasmid. Three candidate crRNAs targeting the *hphS* gene were then designed and screened for cleavage activity. Among the tested crRNAs, crRNA1 produced the strongest fluorescence signal and was therefore selected for subsequent experiments (Figure 3A,B). Through checkerboard titration, the optimal concentrations were established as 1 µM for Cas12a protein and 1 µM for crRNA (Figure 3C). In addition, 5 µM of ssDNA fluorescent probe was selected as the optimal probe concentration following a series of dilution experiments (Figure 3D). Time-course analysis revealed that the fluorescence signal reached a plateau after 20 min of RPA (Figure 3E), and the CRISPR/Cas12a cleavage reaction stabilized after 20 min (Figure 3F). Collectively, the final optimized assay conditions were defined as 1 µM Cas12a, 1 µM crRNA, 5 µM ssDNA reporter, and a 40 min incubation at 37 °C.

### 3.3. Sensitivity of the RPA-CRISPR/Cas12a Fluorescence Assay

**Figure 4:**
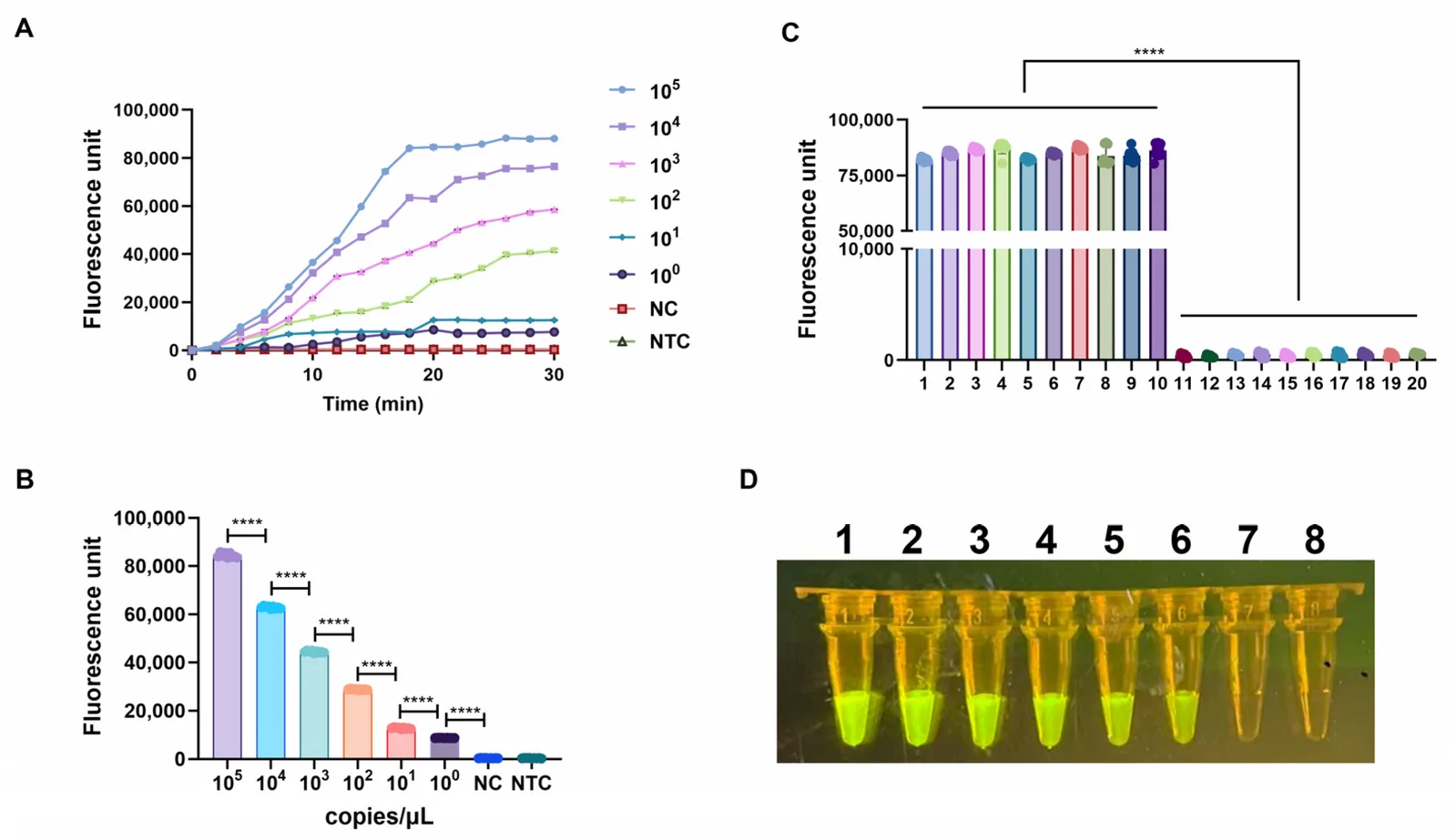
Sensitivity of the RPA-CRISPR-Cas12a detection system. (**A**) Fluorescence curves for the detection of serial 10-fold dilutions of the pMD-19T-*hphS* plasmid standard. (**B**) Fluorescence intensity of the RPA-CRISPR/Cas12a assay measured at 20 min. NC: negative control. (**C**) The LOD was further validated by independent replicate test. 1–10: Plasmid concentration is 1 copy/μL; 11–20: Plasmid concentration is 0 copy/μL. (**D**) Visual fluorescence readout under a portable blue light transilluminator. Lanes 1–6 correspond to plasmid concentrations of 10^5^, 10^4^, 10^3^, 10^2^, 10^1^, and 10^0^ copies/μL, respectively; lane 7 is the NC; lane 8 is the NTC. Data represent mean ± SD from three biological replicates, each with three technical replicates. **** *p* < 0.0001. NC: negative control; NTC: no-template control.

To determine the sensitivity of the developed platform, 10-fold serial dilutions of pMD-19T-*hphS* plasmid (ranging from 10^5^ to 10^0^ copies/μL) were subjected to the RPA-CRISPR-Cas12a fluorescence assay. Real-time fluorescence analysis revealed a positive correlation between signal intensity and plasmid concentration across the entire 10^0^–10^5^ copies/μL range (Figure 4A). The LOD of the RPA-CRISPR/Cas12a fluorescence method was 1 copy/μL (Figure 4B). To statistically validate this LOD, we performed 10 independent replicate tests at the concentration of 1 copy/μL, alongside 10 negative controls. The detection rate at 1 copy/μL was 100%, while all negative controls remained negative, confirming a ≥95% detection probability at this concentration (Figure 4C). With detection performed on a blue light transilluminator, the limit of detection was determined to be 1 copy/μL, where unambiguous fluorescence was still readily discernible (Figure 4D).

### 3.4. Specificity of the Rpa-Crispr-Cas12a Fluorescence Assay

**Figure 5:**
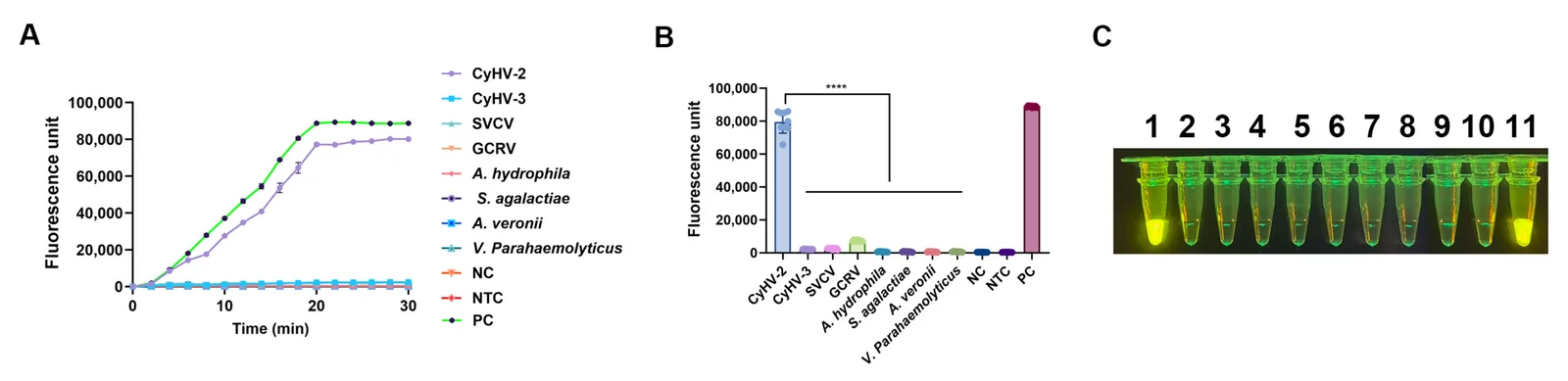
Specificity of the RPA-CRISPR-Cas12a detection system. (**A**) Fluorescence curves of the RPA-CRISPR/Cas12a assay using nucleic acids extracted from CyHV-2, CyHV-3, SVCV, GCRV, *Aeromonas hydrophila*, *Streptococcus agalactiae*, *Aeromonas veronii* and *Vibrio Parahaemolyticus*. (**B**) Endpoint fluorescence intensity of the assay tested against nucleic acids extracted from CyHV-2, CyHV-3, SVCV, GCRV, *Aeromonas hydrophila*, *Streptococcus agalactiae*, *Aeromonas veronii* and *Vibrio Parahaemolyticus*. (**C**) Visual fluorescence detection under a portable blue light transilluminator. 1–8: the genomes of CyHV-2, CyHV-3, SVCV, GCRV, *A. hydrophila*, *S. agalactiae*, *A. veronii*, and *V. parahaemolyticus*, respectively. 9: NC; 10: NTC; 11:PC. Data represent mean ± SD from three biological replicates, each with three technical replicates. **** *p* < 0.0001. PC: positive control (pMD-19T-*hphS* plasmid); NC: negative control; NTC: no-template control.

The specificity of the developed assay was evaluated by testing nucleic acids extracted from CyHV-2 and seven other aquatic pathogens, including CyHV-3, SVCV, GCRV, *Aeromonas hydrophila*, *Streptococcus agalactiae*, *Aeromonas veronii* and *Vibrio parahaemolyticus*. The results showed that a strong fluorescent signal was produced only for CyHV-2, while the other species showed no fluorescence (Figure 5A,B). Under blue light, only the CyHV-2-positive sample exhibited a green fluorescence, while all other pathogens showed no detectable fluorescence (Figure 5C).

### 3.5. Clinical Sample Validation

**Figure 6:**
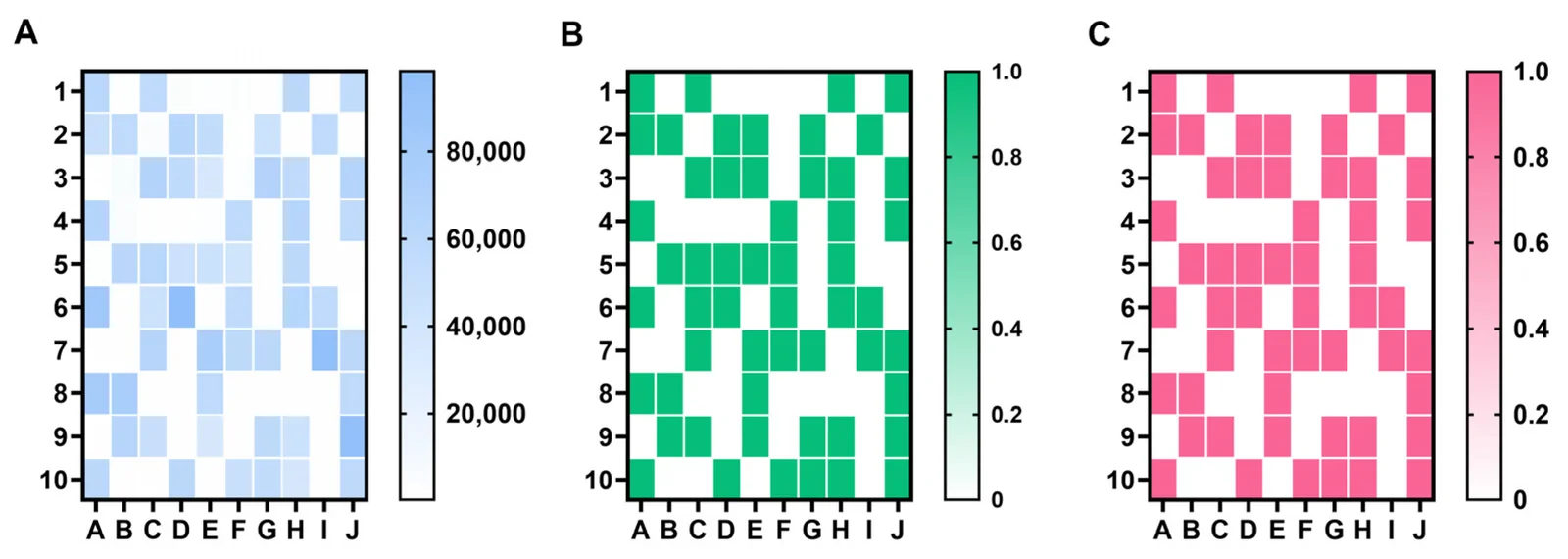
Clinical sample validation of the RPA-CRISPR-Cas12a detection system. (**A**) Fluorescence detection results of 100 clinical samples (representative of three independent experiments). Darker color indicates a stronger fluorescence signal of the samples. (**B**) Visual fluorescence detection results of 100 clinical samples (representative image from one of three independent experiments). The color intensity represents the relative fluorescence signal strength. (**C**) Results of qPCR assay targeting *hphS* in 100 clinical samples (representative of three independent experiments). Positive samples were marked in Rose pink and negative samples were marked in white. Clinical sample IDs are labeled on the left and bottom sides of the figure.

To evaluate the clinical diagnostic performance of the RPA-CRISPR-Cas12a fluorescence detection method, 100 tissue samples from diseased goldfish and crucian carp were tested in parallel using the developed method and a validated qPCR assay. The results from the fluorescence microplate reader (Figure 6A) and the visual fluorescence detection (Figure 6B) were in complete agreement with the qPCR data (Figure 6C), correctly identifying all 50 diseased positive samples, all 4 asymptomatic carrier positive samples, and all 46 healthy negative samples, yielding 100% concordance. The entire testing process, from nucleic acid extraction to final visual fluorescence readout, was completed within approximately 1 h, substantially faster than conventional qPCR (typically requiring 2–3 h).

## 4. Discussion

CyHV-2 represents an emerging threat to cyprinid aquaculture with significant implications for the ornamental fish trade and food fish production [19]. In high-density farming environments, the rapid spread of CyHV-2 highlights the urgent need to develop tools capable of conducting on-site rapid and accurate diagnoses [20]. Traditional diagnostic methods like virus isolation, serology, and PCR-based assays have been helpful for CyHV-2 diagnosis [7,21,22]. However, they have obvious drawbacks, such as long processing time, high equipment requirements, and high cost, which limit their application in clinical settings. The development of isothermal amplification techniques, such as RPA and LAMP, combined with CRISPR-based detection systems, offers new options. These methods are more portable and easier to operate, thus being more practical in molecular diagnosis [23,24,25].

In this study, we developed an RPA-CRISPR-Cas12a fluorescence-based platform for visual detection of CyHV-2. This system combines the rapid isothermal amplification capability of RPA, the high specificity of CRISPR-Cas12a-mediated target recognition, and the simplicity of fluorescence-based visual readout using a portable blue light transilluminator. The detection limit reached 1 copy/μL, which is comparable to or superior to previously reported CyHV-2 detection methods. Our platform achieves higher sensitivity mainly because of two factors. First, RPA pre-amplification enhances the target signal. Second, the collateral cleavage activity of Cas12a provides an additional round of signal amplification. Together, they allow detection even when the template concentration is extremely low.

The specificity of the assay was validated against other common aquatic pathogens, including CyHV-3, SVCV, GCRV, *Aeromonas hydrophila*, *Streptococcus agalactiae*, *Aeromonas veronii* and *Vibrio Parahaemolyticus*, with no cross-reactivity observed. This is critical for reliable clinical diagnostics in aquaculture, where multiple pathogens often co-circulate. However, we acknowledge that the current specificity verification still fails to cover all possible co-infecting pathogens or diverse CyHV-2 isolates. Future work will include a more comprehensive specificity panel, covering more pathogens in various aquaculture environments, to further enhance the diagnostic reliability. To better demonstrate the advantages of our RPA-CRISPR/Cas12a platform, we compared its performance with other commonly used methods for CyHV-2 or related aquatic pathogens. Compared with traditional PCR, our method does not require a thermal cycling device and the detection time can be shortened to within one hour [26]. When compared with RPA-alone detection, the incorporation of CRISPR/Cas12a collateral cleavage provides additional sequence-specific recognition that effectively distinguishes non-specific amplification products, thereby substantially enhancing diagnostic specificity and reducing false-positive rates [27]. Relative to LAMP-based methods, our system is more convenient in designing primers, and the results are more intuitive and clear [23,25]. Among CRISPR-based diagnostic platforms, our system is distinct from SHERLOCK and DETECTR in that it integrates RPA pre-amplification with Cas12a detection in a closed one-tube format and employs direct fluorescence visualization under a portable blue light transilluminator, without the need for multiple transfer steps or expensive fluorescence readers [12,13].

A key innovation of our platform is the fluorescence-based visual readout system using a portable blue light transilluminator. Compared to lateral flow dipstick (LFD) methods, our approach is simpler. LFD requires multiple transfer steps and careful reading of test line intensity. In contrast, the fluorescence detection provides a more straightforward “yes/no” visual readout. The fluorescence signals can be directly observed in the reaction tubes without any additional processing steps, making the operation process simpler and reducing the risk of contamination. To ensure the objectivity of the assessment, we implemented independent evaluation by two trained and blinded observers to minimize subjectivity. In rare cases, if there was a disagreement between the observers, a third researcher would intervene and make the final judgment based on the majority opinion. However, we also acknowledge that when weak positive signals are present near the detection limit, there is still a risk of bias in visual interpretation. In the future, we plan to develop an automated fluorescence analysis application based on smartphones. This application can objectively and quantitatively read the fluorescence intensity from the captured images, further reducing observer bias and enabling remote data sharing and tele-diagnosis in resource-limited settings.

It should be noted that this method still has several limitations. For instance, it still requires a nucleic acid extraction step. If a direct sample detection method without nucleic acid extraction can be developed, it will significantly enhance its applicability in practical applications [28]. Additionally, these samples are insufficient to comprehensively evaluate the sensitivity and specificity of the diagnosis and cannot cover all the field conditions including different geographical regions, seasons, fish species, and co-infecting pathogens. Therefore, we are currently collecting over 300 clinical samples from multiple fish farms across three provinces in China (Jiangsu, Hubei, and Guangdong), covering different fish species such as common carp, goldfish, and other cyprinids. This large-scale validation will allow us to further assess the robustness and reliability of this platform under diverse field conditions. Moreover, although our in silico sequence alignment confirmed that the *hphS* gene is highly conserved across all currently available CyHV-2 genotypes (including J genotype and C genotype), with no mismatches in primer or crRNA binding sites, we recognize that emerging variants or recombinant strains may arise. Therefore, it is necessary to regularly re-evaluate the specificity of primers and crRNAs for new isolates to ensure the long-term usability of this diagnostic platform. In addition, although our system adopts a single-tube design and includes multiple negative controls, which significantly reduces the risk of false positives caused by amplification residue, we still need to acknowledge that the high amplification efficiency of RPA still requires strict pollution control measures, especially in field applications. To further alleviate this concern, future iterations of the assay could incorporate uracil-DNA glycosylase (UNG) and deoxyuridine triphosphate (dUTP) in the RPA reaction to enzymatically degrade any carryover amplicons, as previously described for other isothermal amplification systems [29]. Additionally, the development of lyophilized, pre-dispensed reagent pellets would minimize pipetting steps and further reduce cross-contamination risks. These enhancements, combined with rigorous physical separation of workflow stages and the use of aerosol-resistant pipette tips, will be essential for ensuring the reliability of this platform under aquaculture conditions. Furthermore, the current platform provides qualitative “yes/no” detection results and does not support quantitative virus load measurement. Although this qualitative result is sufficient for rapid on-site screening in resource-limited aquaculture environments, we acknowledge that the lack of quantitative capability may limit its application in scenarios where monitoring the severity of infection, disease progression, or treatment efficacy is required. To address this limitation, future research will establish a standard curve that can correlate fluorescence intensity with template copy number under strictly controlled reaction conditions, thereby enabling semi-quantitative or even quantitative analysis.

In summary, we have successfully developed and validated a single-tube fluorescence detection method based on RPA and CRISPR-Cas12a for the detection of CyHV-2. This method is rapid, sensitive, highly specific, and enables visual detection. The method is simple to operate, low in cost, and compatible with various instruments, making it suitable for on-site detection in various aquaculture environments. The successful application of this technology will enhance the monitoring and prevention capabilities of CyHV-2, especially being of great significance in regions with limited resources.

## 5. Conclusions

In this study, we successfully established a one-tube RPA-CRISPR-Cas12a-based fluorescence platform for the rapid and visual detection of CyHV-2. The assay reached a detection limit of 1 copy/μL. It showed no cross-reactivity with other common aquatic pathogens. In clinical samples, it achieved 100% concordance with qPCR. These results indicate that this platform can serve as a simple, low-cost and flexible tool for on-site monitoring of CyHV-2, especially suitable for aquaculture environments with limited resources.

## Figures and Tables

**Table 1:** Primers and crRNAs used in this study.

Primer	Sequence (5′→3′)	Function
F1	GATCAATCACAATAACGTGCAGAACCCGGA	RPA primers
R1	AACCATAGTCACCATCGTCTCATCAAACAA	
F2	ACAATAACGTGCAGAACCCGGACGAGCGTA	
R2	GTCTCATCAAACAAACAAACAAACAAAAAA	
F3	ATCTTCAAGCCCAAGATCAATCACAATAAC	
R3	ACAAACAAACAAACAAAAAAAGGCGTACCA	
*hphS* *-F*	TTTATTGAGAGAGGCGGC	Construction of pMD-19T-*hphS* recombinant plasmid
*hphS* *-R*	CAACTCGGTTTGATGGGA	
crRNA1	UAAUUUCUACUCUUGUAGAUCAGCCGUGGUCCGUCAACGGUUCAAGCACA	CRISPR RNA for Cas12a detection
crRNA2	UAAUUUCUACUCUUGUAGAUAAGGCGUCCGAAUCAAGGUCGGAUCU	
crRNA3	UAAUUUCUACUCUUGUAGAUAAAGAUGCGAGCAGGCCCAGAACGUG	
*hphS*-qPCR-*F*	TTTCTACACGCCTCGCATCAT	Detection of the *hphS* gene
*hphS*-qPCR-*R*	CCCAGACAGCCTTCAAACACT	

## Data Availability

The original contributions presented in this study are included in the article/Supplementary Materials.

## Supplementary Materials

The following supporting information can be downloaded at: https://www.mdpi.com/article/10.3390/vetsci13090970/s1, Figure S1. The positions of RPA primers and crRNAs within the *hphS* gene.
