# Supplementary material for: Rapid Visual Detection of Cyprinid Herpesvirus 2 Based on One-Tube RPA-CRISPR/Cas12a System
Source: Vet Sci. 2026 Sep 16;13(9):970. doi: 10.3390/vetsci13090970 (PMC13611860; doi:10.3390/vetsci13090970)
Supplement: Supplementary file 1 [file vetsci-13-00970-s001.zip › vetsci-4553403-supplementary/vetsci-4553403-supplementary.pdf]

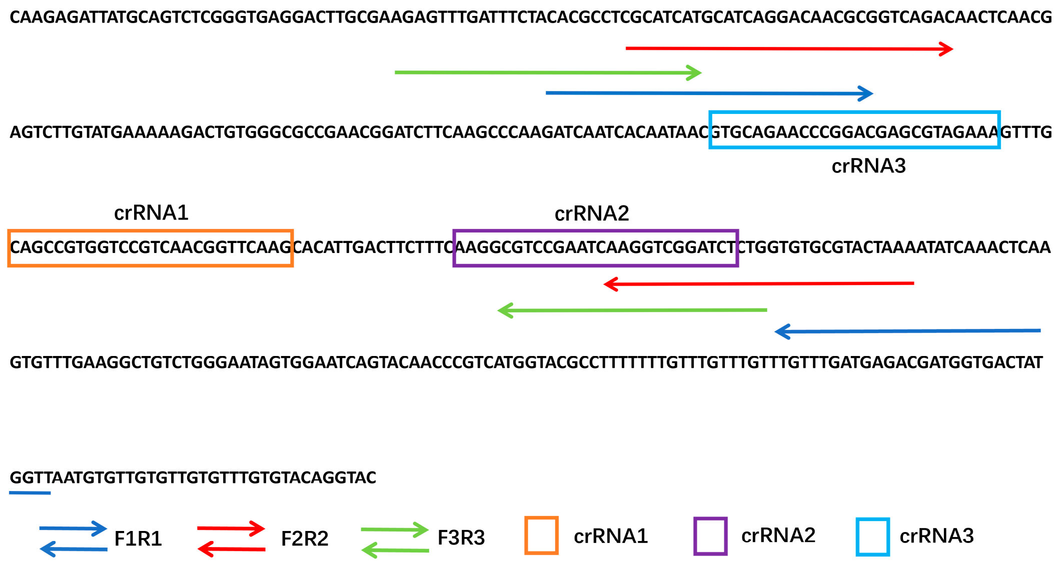

Figure S1. The positions of RPA primers and crRNAs within the *hphS* gene.
